# A tale of two sequences: microRNA-target chimeric reads

**DOI:** 10.1186/s12711-016-0209-x

**Published:** 2016-04-04

**Authors:** James P. Broughton, Amy E. Pasquinelli

**Affiliations:** Division of Biology, University of California, San Diego, La Jolla, CA 92093-0349 USA

## Abstract

In animals, a functional interaction between a microRNA (miRNA) and its target RNA requires only partial base pairing. The limited number of base pair interactions required for miRNA targeting provides miRNAs with broad regulatory potential and also makes target prediction challenging. Computational approaches to target prediction have focused on identifying miRNA target sites based on known sequence features that are important for canonical targeting and may miss non-canonical targets. Current state-of-the-art experimental approaches, such as CLIP-seq (cross-linking immunoprecipitation with sequencing), PAR-CLIP (photoactivatable-ribonucleoside-enhanced CLIP), and iCLIP (individual-nucleotide resolution CLIP), require inference of which miRNA is bound at each site. Recently, the development of methods to ligate miRNAs to their target RNAs during the preparation of sequencing libraries has provided a new tool for the identification of miRNA target sites. The chimeric, or hybrid, miRNA-target reads that are produced by these methods unambiguously identify the miRNA bound at a specific target site. The information provided by these chimeric reads has revealed extensive non-canonical interactions between miRNAs and their target mRNAs, and identified many novel interactions between miRNAs and noncoding RNAs.

## Background

### Target recognition by miRNAs

MicroRNAs (miRNAs) are an important class of regulatory molecules that function to target specific RNAs for posttranscriptional regulation [1]. Prevalent in animals and plants, miRNAs are small (~22 nucleotides), noncoding RNAs (ncRNAs) that bind to Argonaute (AGO) proteins. Once the miRNA is bound to Argonaute, as part of the miRNA induced silencing complex (miRISC), it guides miRISC to target RNAs. In animals, these target sites are usually located in the 3′ untranslated region (3′UTR) of the mRNA, but may also reside within the coding sequence or 5′UTR. Protein production from mRNAs that are targeted by miRNAs is subsequently repressed due to inhibition of translation and transcript destabilization.

In animals, miRNAs interact with their targets through imperfect base pairing. The limited sequence interactions required by miRNAs to direct regulation allows a single miRNA to potentially regulate hundreds of targets in multiple pathways. Although miRNAs are flexible in their targeting ability, a large body of work has proposed a series of rules that predict canonical miRNA targeting [2, 3]. Nucleotides 2–8 at the 5′ end of the miRNA are known as the seed sequence and are important for miRNA target recognition. Crystal structures of miRNAs bound to Argonaute proteins have suggested that the seed sequence is favorably positioned to initiate the interaction between miRNAs and their target RNAs [4–6].

Perfect seed complementarity defines canonical targeting, but there are a variety of examples of imperfect or non-seed interactions [2]. However, the extent to which miRNAs interact with their targets non-canonically and whether these targets are functional remains unclear [7]. In addition, recent evidence has suggested that miRNAs may have functional interactions with other ncRNAs [8–10]. The prevalence of these interactions is an open question.

### Challenges in the identification of miRNA targets

The identification of miRNA target sites remains an outstanding challenge. In particular, pinpointing miRNA target sites is complicated due to the small size of miRNAs and their ability to functionally interact with their targets through imperfect base pairing. These two constraints limit the sequence information that can be used to predict targets, while also allowing a single miRNA to potentially regulate many targets.

Various research groups have developed computational approaches to predict miRNA target sites. For example, the commonly cited TargetScan algorithm was originally designed to predict target sites by looking for seed sequence complementarity and conservation in 3′UTRs [11, 12]. However, computational prediction programs are generally limited by the current understanding of miRNA targets and may miss unexpected functional interactions, such as those between a miRNA and another noncoding RNA [8]. In addition, comparisons of miRNA target prediction algorithms show that there is limited overlap between the targets that are predicted by various programs [13]. This suggests that many targets that are identified by current miRNA target prediction algorithms are false positives. In recent years, bioinformatics approaches have improved by taking into consideration additional information, including the binding sites of Argonaute proteins and the secondary structure of the target site [7, 14].

In addition to computational prediction programs, functional RNA interference (RNAi) assays have also been used to identify miRNA targets in *Caenorhabditis elegans*. However, RNAi screens are only able to identify targets that are important for the phenotype of interest and may identify indirect targets. In *C. elegans*, the majority of single miRNA knockouts do not have an observable phenotype [15], as a consequence, the use of RNAi screens to detect targets that are regulated by miRNAs can be misleading. However, in other organisms, screens that inhibit miRNA targets may be more useful. For example, in *Drosophila melanogaster* more than 80 % of the miRNA mutants, 20 % of which have mutations in multiple miRNAs, have abnormal phenotypes [16].

Other approaches to identify miRNA targets have focused on quantifying protein or RNA levels of candidate genes. Techniques applied to the identification of miRNA targets include stable isotope labeling by amino acids in cell culture (SILAC) [17–19] and ribosome profiling [20, 21]. These approaches can be biased by the selection of candidate targets and may reveal indirect targets [1]. Furthermore, the analysis of gene expression changes after altered miRNA levels does not identify the specific target site of the miRNA.

Recently, the identification of Argonaute binding sites through crosslinking immunoprecipitation (CLIP) based methods, such as CLIP-seq (cross-linking immunoprecipitation with sequencing), PAR-CLIP (photoactivatable-ribonucleoside-enhanced CLIP), and iCLIP (individual-nucleotide resolution CLIP), has increased the understanding of how miRNAs interact with their target sites [22–25]. In general, CLIP-based methods identify protein-RNA binding sites by crosslinking proteins to interacting RNA molecules, purifying these protein-RNA complexes, and sequencing the associated RNAs. Although CLIP-based approaches define the region of an RNA that an Argonaute protein is bound to, these methods do not specifically identify the miRNA that is responsible for the identified interaction [1]. This is problematic for families of miRNAs that share the same seed sequence, for sites that contain seed complementarity to multiple miRNAs, or for sites with no obvious pairing to known miRNAs.

## Review

### Ligation of two RNA molecules identifies RNA–RNA interactions

**Fig. 1:**
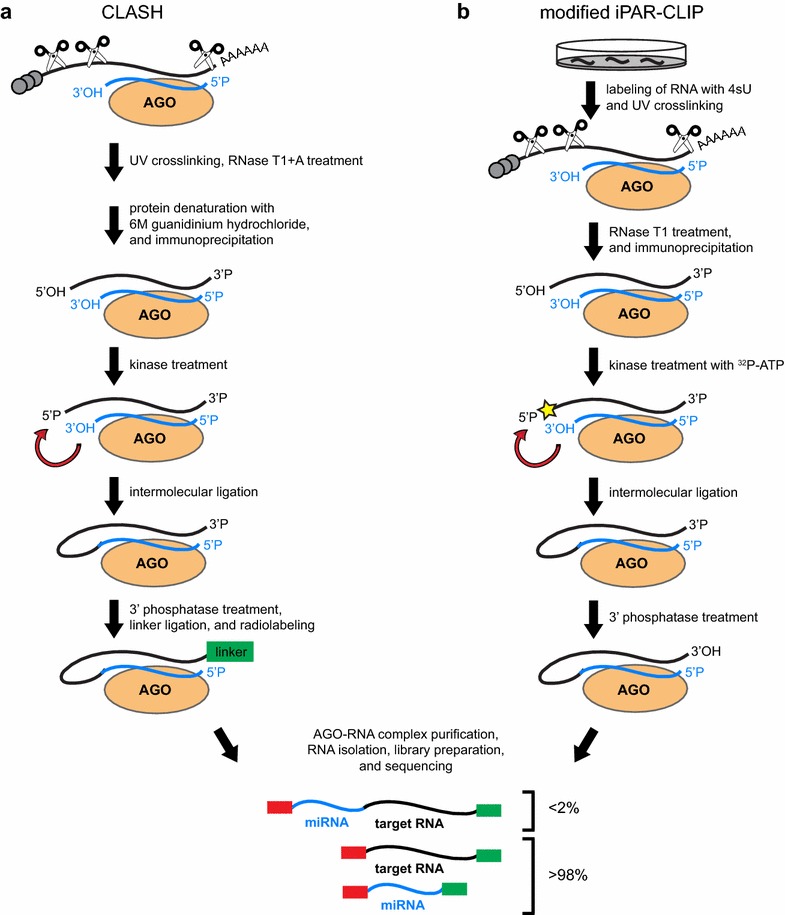
**a** Overview of CLASH and **b** modified iPAR-CLIP methods for the formation of miRNA-target chimeras. CLASH begins with trimming of unprotected RNAs in UV crosslinked lysates with RNase and denaturation of the AGO-miRNA-target RNA tertiary complex. In modified iPAR-CLIP, the sample (*C. elegans* worms, for example) must be incubated with 4-thiouridine (4sU) for RNA incorporation to enhance UV crosslinking. Both CLASH and modified iPAR-CLIP protocols phosphorylate the 5′ end of the target RNA, which is then ligated to the miRNA using an exogenous RNA ligase. Subsequent 3′ end phosphatase treatment prepares the RNA for linker ligation. In CLASH, the 3′ linker is added during the “on-bead” biochemical steps, whereas in modified iPAR-CLIP, the 3′ linker is added after RNA isolation. The majority of the reads generated from CLASH and modified iPAR-CLIP are not chimeric

Whereas CLIP-based methods are able to identify protein-RNA interaction sites, RNA–RNA interaction sites can be identified by crosslinking and sequencing of hybrids (CLASH) and similar approaches [26–28]. Akin to CLIP-seq, CLASH involves the purification and sequencing of crosslinked protein-RNA complexes. However, in CLASH, additional biochemical steps promote the intermolecular ligation of RNA molecules to form a hybrid, or chimeric, read composed of two RNA molecules (Fig. 1a).

CLASH was developed after the observation that chimeric reads occurred in crosslinking and analysis of cDNAs (CRAC) data. These hybrid reads were not the product of reverse transcriptase template switching, and were likely generated as a result of the step in CRAC that ligates oligonucleotide linkers to RNA [26]. The first application of CLASH was the identification of snoRNA target sites on pre-rRNAs in yeast from C/D snoRNA-associated proteins. From the sequencing library generated by CLASH for these proteins, 0.1–0.8 % of the reads were chimeric. The majority (74 %) of snoRNA-pre-rRNA chimeric reads produced from this application of CLASH recovered known target sites. However, some reads identified potentially novel snoRNA-pre-rRNA sites. Other chimeric reads from Kudla et al. [26] produced rRNA–rRNA reads, which were thought to be nonspecific interactions.

The ability of CLASH to identify RNA–RNA interactions was subsequently applied to the identification of AGO1 miRNA target sites in human embryonic kidney 293 (HEK293) cells [27]. From the AGO1 CLASH data, 98 % of the reads were not chimeric and contained sequence information similar to that produced by CLIP-seq. The remaining 2 % of CLASH data contained chimeric reads and were composed of the mature miRNA sequence ligated to a target RNA molecule. In 69.8 % of the miRNA chimeras, the target RNA mapped to mRNAs. Additional RNAs that were found to be ligated to mature miRNAs included other miRNAs, rRNAs (ribosomal RNAs), tRNAs (transfer RNAs), pseudogenes, and lincRNAs (long intergenic non-coding RNAs). By including a control in which yeast total RNA was mixed with the cell lysates before carrying out the CLASH protocol, Helwak et al. [27] demonstrated that less than 2 % of CLASH chimeric reads were nonspecific. Thus, these non-mRNA targets identified by CLASH may be examples of miRNA interactions with non-coding RNAs.

An alternative approach for the generation of chimeric reads was developed by including an intermolecular ligation step in iPAR-CLIP [28]. This modified version of iPAR-CLIP produced chimeric reads in *C. elegans* in a similar manner to CLASH (Fig. 1b). Based on the sequencing data produced by modified iPAR-CLIP, 0.24 % of the reads were miRNA-target chimeras. As with CLASH, the chimeric reads appear to be highly specific with less than 2 % of the reads mapping to background bacterial sequences and 92 % of the chimeric reads mapping to mRNAs.

### miRNA-target chimeras from standard CLIP-seq library preparation

**Fig. 2:**
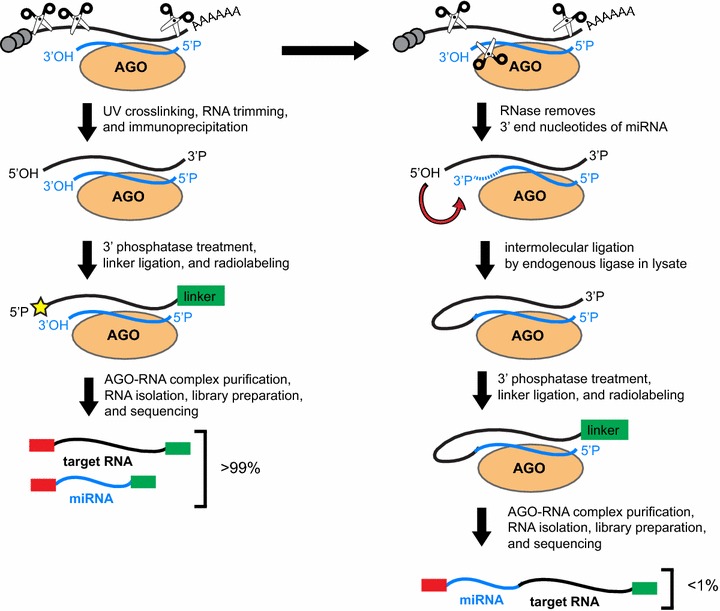
Comparison of the biochemical steps in CLIP-seq for the generation of standard CLIP-seq reads to events that can lead to the formation of miRNA-target chimeric reads in CLIP-seq or iPAR-CLIP. Standard CLIP-seq reads are generated after RNA trimming of UV crosslinked lysates and immunoprecipitation of the AGO-miRNA-target RNA tertiary complex. The 3′ end of the RNA is then prepared for linker ligation and the complex is radio-labeled to facilitate the isolation of the complex. Chimeric reads may form in CLIP-seq when partial digestion of the 3′ end of the miRNA by RNase during the RNA trimming step of CLIP-seq or iPAR-CLIP produces a 2′–3′ cyclic phosphate or a 3′ phosphate. Endogenous ligases in the lysate have been predicted to be responsible for ligation of the 3′ end of the digested miRNA to the 5′ phosphate of the target RNA. The subsequent steps that occur in the CLIP-seq protocol prepare the miRNA-target chimera for sequencing

Grosswendt et al. [28] also found that chimeric reads were generated in iPAR-CLIP libraries that did not contain the additional intermolecular ligation step. This finding was surprising because standard iPAR-CLIP is not designed to produce the correct 5′ and 3′ end chemistry to allow for intermolecular ligations between miRNAs and target RNAs. However, the authors noticed that the chimeras produced by standard iPAR-CLIP tended to include a truncated miRNA sequence. They therefore concluded that the RNA trimming step in iPAR-CLIP was responsible for generating the ligated products. Specifically, they predicted that RNase T1 was partially trimming the 3′ end of the miRNA producing a 2′–3′-cyclic phosphate, which could then be ligated to the 5′ hydroxyl of the target RNA through the action of endogenous ligases present in the lysate (Fig. 2). The production of chimeras was less efficient in standard iPAR-CLIP than in the modified iPAR-CLIP (which included an exogenous ligase to catalyze intermolecular ligations), with only 0.16 % of reads being miRNA-target chimeras. Using this information, Grosswendt et al. [28] reanalyzed previously published CLIP-seq and PAR-CLIP data from human and mouse and found approximately 13,000 additional miRNA-target chimeras.

### Bioinformatic identification of miRNA-target chimeric reads

Chimeric reads were identified similarly in CLASH and modified iPAR-CLIP. In both cases, duplicate reads and adapter sequences were removed before identifying chimeric reads. In CLASH, non-contiguous reads were identified using BLAST against transcriptome databases, tRNA, rRNA, and mature miRNA sequences [27, 29]. Non-contiguous reads that contained a miRNA sequence were considered miRNA chimeras. Grosswendt et al. [28] searched reads from modified iPAR-CLIP for all possible 12 nucleotide sequences from mature miRNAs to identify putative miRNA-target chimeras [28]. The identity of the miRNA was then assigned by aligning the read to the full-length miRNA sequence. The method applied in [28] guaranteed that truncated miRNAs or miRNA reads with mutations would also be recovered.

To ensure that the entire target site was identified, both Helwak et al. [27] and Grosswendt et al. [28] computationally increased the size of the recovered target sequence. In CLASH, the target sequence in the chimeric read was increased by 25 nucleotides. The reads from the modified iPAR-CLIP method were increased by 8 nucleotides upstream and 12 nucleotides downstream. These adjustments helped to increase the number of seed matches with the target RNA and facilitated clustering of overlapping target sequences to identify miRNA target sites [27, 28].

### Insights from miRNA-target chimeric reads

Although miRNAs are known to primarily direct Argonaute proteins to the 3′UTR of target mRNAs, many target sites that are identified by CLASH (42.6 %) and modified iPAR-CLIP (23.4 %) are located in coding exons. Similarly, Argonaute binding sites have been identified in coding exons nearly as frequently as in 3′UTR from CLIP-seq and PAR-CLIP datasets [22–24]. Complementarity to miRNA seed sequences has been observed in coding exons, but the functionality of these potential target sites remains unclear. In *C. elegans*, transcripts with coding exon Argonaute binding sites generally did not appear to be deregulated after the loss of Argonaute, whereas transcripts with 3′UTR binding sites were [23]. Similarly, transcripts with coding exon target sites of human Argonaute identified by PAR-CLIP in human embryonic kidney 293 (HEK293) cells were not as strongly regulated as target sites in 3′UTR [22]. Some studies have shown that coding sequence targets function cooperatively with 3′UTR targets to enhance regulation [30], whereas others have suggested that these target sites promote translational inhibition rather than mRNA stabilization [31].

Since chimera-producing methods are able to identify both the miRNA and the target site, it is possible to classify the types of miRNA-target interactions that occur. Helwak et al. [27] applied *k*-means clustering to identify five classes of miRNA-target interactions from 18,514 miRNA-mRNA chimeras. These classes included seed only, seed with 3′ supplementary (nucleotides 13–16) pairing, seed with terminal 3′ end pairing, non-seed, and dispersed interactions. Targets with seed and seed with supplementary interactions were the most efficient at down-regulating targets and were the most conserved. Interestingly, 45 % of miRNAs appeared to have nonrandom types of interactions with their targets, i.e. some miRNAs preferentially binding to seed sites and other miRNAs having more extensive non-seed interactions. Overall, only 37 % of the miRNA-mRNA chimeras identified from the CLASH data contained perfect seed matches.

Grosswendt et al. [28] also looked at the prevalence of seed interactions in modified iPAR-CLIP data and found that 43 % of the targets had perfect seed matches with their targets. However, when they included near-seed matches, such as one nucleotide mismatch and one nucleotide bulge, 80 % of the *C. elegans* chimeras contained seed interactions. In contrast to the many non-seed interactions that were identified by CLASH, Grosswendt et al. [28] observed limited evidence for 3′ end interactions in the 3627 chimeras they examined from *C. elegans*. Similarly, the ~13,000 chimeras that were identified from traditional CLIP-seq and PAR-CLIP datasets also showed limited non-seed interactions.

### miRNA-target chimeras identify non-canonical target sites

The CLASH-generated chimeras suggest that ~60 % of the identified target sites were non-canonical with imperfect or non-seed interactions. To test whether non-canonical target sites for miR-92a were functional, Helwak et al. [27] generated reporter constructs that contained miR-92a seed sites, miR-92a 3′ end interaction motifs, and a combination of both seed and 3′ end interaction motifs. For each of these three constructs, inhibition of miR-92a led to deregulation of the reporter. However, the construct that contained only the miR-92a 3′ end interaction motif was only moderately deregulated after miR-92a knockdown.

Recently, RNA expression datasets were independently analyzed for the regulation of miR-92a CLASH identified targets. In one dataset of miR-92a knockdown HEK293 cells, both canonical and non-canonical miR-92a target genes were significantly deregulated [7]. Although the non-canonical targets were deregulated, this effect was not particularly strong in comparison to the canonical targets. To further explore whether these non-canonical targets are functional, expression data from the knockdown of 25 miRNAs, including miR-92a, was analyzed. In this dataset, the canonical miR-92a targets identified by CLASH were significantly deregulated, whereas the non-canonical miR-92a targets were not [7]. In addition, Agarwal et al. [7] examined the expression of non-canonical targets that were identified by CLASH for four miRNA families and observed that these non-canonical sites were not significantly deregulated, even if the site occurred within a 3′UTR. The minimal regulation in the miR-92a non-canonical reporters that was observed by Helwak et al. [27] and the analysis of RNA expression in miRNA knockdowns that was conducted by Agarwal et al. [7] suggest that these non-canonical target sites are either not nearly as functional as canonical seed-containing target sites or may not be functional at all.

In addition to non-canonical seed interactions, many of the chimeras identified by CLASH and modified iPAR-CLIP targeted ncRNAs, such as tRNAs, other miRNAs, and lincRNAs. Due to the low level of background ligation events with yeast RNA (CLASH) and bacterial RNA (modified iPAR-CLIP), it is likely that many of these interactions are specific. Although the biological significance of most of these miRNA-ncRNA interactions remains to be determined, Helwak et al. [27] demonstrated that the inhibition of a miRNA targeting a lincRNA resulted in the up-regulation of the lincRNA. This indicates a functional role for some miRNA-ncRNA interactions. Competing endogenous RNAs (ceRNAs) have been proposed to sequester miRNAs from their targets [9, 10]. However, recent analysis of ceRNAs has suggested that, at physiological levels, many ceRNAs may not be sufficiently highly expressed to effectively sequester miRNAs [25]. Helwak et al. [27] propose that the prevalence of chimeras that map to rRNAs and tRNAs implies that these abundant RNAs may also have a role in sequestering miRNAs from their targets.

## Conclusions

miRNA-target chimeric reads provide unambiguous determination of the identity of a miRNA that is bound at a target site, whereas previously it had to be assumed from seed complementarity or other features. In addition to correctly assigning miRNAs to their endogenous target sites, chimeras allow for detailed analysis of the types of interactions that miRNAs have with their targets. The extensive non-canonical interactions identified by CLASH may provide insights into how miRNAs choose their targets in vivo. While this article was in review, a new report on the analysis of miRNA-target chimeras concluded that pairing to miRNA 3′ end sequences is more important than previously considered [32]. In addition to patterns of hybridization with targets, analysis of chimeric sites may reveal features that explain why certain 3′UTRs are predominantly regulated by a single miRNA despite seed complementarity to other expressed miRNAs. Furthermore, chimeras allow the identification of ncRNA targets of miRNAs. These interactions with ncRNAs may be transient but still have biological importance.

Although miRNA-target chimeric reads are a unique tool to understand miRNA targeting, there are still several limitations to the current protocols. Foremost is the low fraction of chimeric reads that are generated by the new methods. With the CLASH and modified iPAR-CLIP methods, 2 and 0.24 % of the libraries were chimeric reads, respectively [27, 28]. As a consequence of the limited number of available reads, many target sites were identified by a single chimera. In the modified iPAR-CLIP data, only 18.7 % of the target sites had more than one read [28]. Given this observation, it is unlikely that CLASH or modified iPAR-CLIP identify the complete set of miRNA-target interactions. Furthermore, it will be important to focus on reproducible chimeras, since isolated examples may represent sampling of targets by miRISC and not authentic targeting. In line with these considerations, a comparison of CLASH chimeric reads to the most recent implementation of TargetScan led to the conclusion that TargetScan is better at predicting functional miRNA targets than the experimentally-derived CLASH chimeras [7]. Future work will need to focus on enriching for chimeras that represent functional targeting events to deepen our understanding of how miRISC chooses appropriate regulatory targets in vivo.
